# Thermal performance of the heterotrophic dinoflagellate *Oxyrrhis marina* under long-term warming and acute heat stress

**DOI:** 10.1093/plankt/fbaf013

**Published:** 2025-08-12

**Authors:** Minerva García-Martínez, Enric Saiz, Albert Calbet

**Affiliations:** Department of Marine Biology and Oceanography, Institut de Ciències del Mar (ICM), CSIC, Passeig Marítim de la Barceloneta 37-49, 08003 Barcelona, Spain; Ph. D. Program on Marine Sciences from the Department of Earth and Ocean Dynamics, Faculty of Earth Sciences, University of Barcelona, Martí i Franquès s/n, 08028 Barcelona, Spain; Department of Marine Biology and Oceanography, Institut de Ciències del Mar (ICM), CSIC, Passeig Marítim de la Barceloneta 37-49, 08003 Barcelona, Spain; Department of Marine Biology and Oceanography, Institut de Ciències del Mar (ICM), CSIC, Passeig Marítim de la Barceloneta 37-49, 08003 Barcelona, Spain

**Keywords:** thermal acclimation, *Oxyrrhis marina*, thermal performance curve, functional response, marine food web

## Abstract

We investigated the thermal tolerance and physiological responses of the heterotrophic dinoflagellate *Oxyrrhis marina* reared for more than 34 months at 16, 19 or 22°C, using *Rhodomonas salina* as prey. First, we constructed the thermal performance curves for the growth rate of the three thermal lines of *O. marina* and found that the maximum growth rates and the optimum temperature increased with higher rearing temperature. Next, we examined the functional responses at each rearing temperature and under acute heat stress (+3°C). Maximum ingestion rates (in cells and carbon) showed limited temperature sensitivity, regardless of chronic or acute heat exposure. Under heat stress, the 16 and 19°C lines saturated at lower prey abundances and showed enhanced clearance rates, whereas the 22°C line experienced negative effects. Growth rates increased with temperature, particularly under acute stress, while growth efficiencies remained stable under long-term thermal exposure and increased only when subjected to acute heat stress. Overall, these findings suggest *O. marina* follows a “Hotter is partially better” model, with notable differences between the 16 and 22°C lines. Our results highlight the importance of considering both gradual and abrupt temperature changes when predicting climate change impacts on marine food webs and biogeochemical processes.

## INTRODUCTION

Herbivorous microzooplankton, mostly ciliates and dinoflagellates, play a crucial role in marine ecosystems due to their significant impact on phytoplankton populations, removing on a global average between 49 and 77% of the planktonic primary production daily (Calbet and Landry, 2004; Schmoker *et al.*, 2013). Additionally, they are key components of the diet of higher trophic level members of the food web, such as copepods (Calbet and Saiz, 2005; Saiz and Calbet, 2011), transferring energy, minerals, vitamins and other substances to them (Klein Breteler *et al.*, 1999; Anderson and Menden-Deuer, 2017). Therefore, variations in microzooplankton abundance, community structure, species interactions, biochemical composition or physiological rates can have severe consequences for the overall functioning of marine ecosystems (Franzè and Menden-Deuer, 2020; Calbet *et al.*, 2022; Ferreira *et al.*, 2022; Franzè *et al.*, 2023). In this regard, two climate change-driven environmental factors expected to impact microzooplankton communities are temperature and prey availability.

It is now evident that the ocean is experiencing an increase in temperature, particularly noticeable in surface waters, but also perceptible at considerable depths (up to 250 m) (Garrabou *et al.*, 2022; CMEMS, 2023). This temperature rise has been reported to affect marine biodiversity by enhancing harmful algae blooms, shifting species composition, disrupting organisms’ life cycles, etc. (Hays *et al.*, 2005; Baltar *et al.*, 2019). Besides the gradual temperature increase, an intensification in the frequency and strength of anomalous episodes such as heat waves is also expected, associated with an increase in mass mortality events worldwide (Hays *et al.*, 2005; Baltar *et al.*, 2019; Garrabou *et al.*, 2022).

**Fig. 1:**
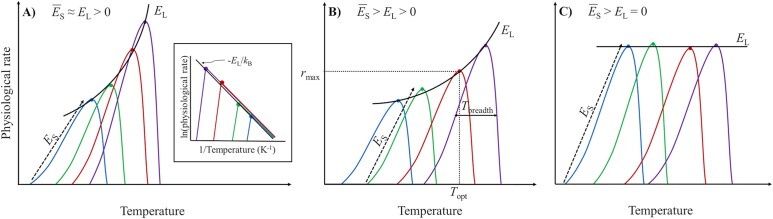
Three possible models of adaptation of organisms for the unimodal response to temperature. Colder-adapted organisms are represented with blue and warmer-adapted with purple. (**A**) “Hotter is better” hypothesis: individuals adapted to warmer temperatures show higher performances than colder-adapted ones. In this hypothesis, the mean (*Ē*_S_) of the activation energies of all thermal performance curves (*E*_S_) is similar to the global activation energy (*E*_L_). Illustration included in chart A represents this hypothesis in the form of the Arrhenius plot, where *k*_B_ is the Boltzmann constant. (**B**) “Partial compensation” or “Hotter is partially better” hypothesis: the performance is enhanced as adaptation temperature rises (*E*_L_ > 0), but individuals do not fully compensate for thermodynamic constraints, so *Ē*_S_ is significantly higher than *E*_L_. (**C**) “Perfect or Complete compensation” hypothesis: the performance is similar in all optimal temperatures and, consequently, *E*_L_ is not different from zero. Points over the curves denote the maximum physiological rate (*r*_max_) at the optimal temperature (*T*_opt_). Chart B also illustrates other thermal performance curve parameters (the maximum rate, *r*_max_; the thermal optimum, *T*_opt_; the amplitude of the curve, *T*_breadth_). Based on Barton and Yvon-Durocher (2019) and Smith *et al.* (2019).

The interaction between temperature and organisms is not straightforward. The response of organisms to temperature is commonly known to be unimodal, with the thermal performance curve of the physiological rate rising exponentially until an optimum where the rate is maximized and then decreasing when the temperature starts to be harmful to the metabolic processes of the organism (Dam *et al.*, 2021). In fact, the metabolism of an organism can be understood as a network of interconnected enzymatic reactions, constrained by thermodynamic principles and responsible for the changes in physiological rates (Frazier *et al.*, 2006; Barton and Yvon-Durocher, 2019; Smith *et al.*, 2019). Traditionally, the activation energy, which represents the energy required for a chemical reaction to occur, has been used to describe the thermal sensitivity of organisms and is known to be related to the thermal environment organisms inhabit (Low *et al.*, 1973; Packard *et al.*, 1975; Luhring and DeLong, 2017). Thus, understanding the activation energy of the different metabolic processes is crucial for evaluating the thermal sensitivity of individuals and their adaptive responses to temperature (Barton and Yvon-Durocher, 2019; Smith *et al.*, 2019; Chen *et al.*, 2023). Generally, the variations in activation energy along the thermal performance curves align with three main hypotheses (Barton and Yvon-Durocher, 2019; Smith *et al.*, 2019; Chen *et al.*, 2023). First, the “Hotter is better” hypothesis postulates that organisms adapted to higher temperatures will experience higher rates and better performances than organisms grown at lower temperatures and that the optimal temperature and maximum performance are positively correlated (Huey and Kingsolver, 1989; Knies *et al.*, 2009). In this case, the mean of the activation energies of the thermal performance curves (*Ē*_S_) is similar to the steepness (i.e. the activation energy) of the rising part of the curve resulting from plotting the optimal temperatures and maximum physiological rates of the performance curves (global activation energy, *E*_L_) (Fig. 1A; Frazier *et al.*, 2006; Barton and Yvon-Durocher, 2019; Smith *et al.*, 2019; Chen *et al.*, 2023). Related to this theory, the “Hotter is narrower” concept would refer to the fact that to achieve a “Hotter is better” model of adaptation, the thermal breadth of the thermal performance curves would be progressively narrower as the optimal temperature and optimal rate increase (Knies *et al.*, 2009). Conversely, the “Hotter is partially better” (also known as “Partial compensation”) hypothesis postulates that the adaptive processes cannot fully compensate for thermal effects, and therefore the average of the activation energies of the thermal performance curves is still significantly higher than the activation energy of the optimal performances (Fig. 1B; Barton and Yvon-Durocher, 2019; Smith *et al.*, 2019; Chen *et al.*, 2023). Finally, the “Perfect or Complete compensation” hypothesis argues that organisms that have overcome thermal selection will have similar performances at their optimal growth temperatures (Fig. 1C; Frazier *et al.*, 2006; Barton and Yvon-Durocher, 2019; Smith *et al.*, 2019; Chen *et al.*, 2023).

Although temperature effects on metabolic processes may change over time and can lead to acclimation and adaptive responses (Havird *et al.*, 2020; de Juan *et al.*, 2023a, 2025), most studies on herbivorous protozoa are based on short-term acclimation responses (e.g. Kimmance *et al.*, 2006; Franzè and Menden-Deuer, 2020; Calbet *et al.*, 2022; Ferreira *et al.*, 2022). In fact, studies on the thermal response of this planktonic group after long-term exposure, allowing for multigenerational processes, are very scarce (Calbet and Saiz, 2022, 2023). Yet, these kinds of studies are of particular interest because global warming appears to be gradual and at a pace that could facilitate adaptive processes (Calbet and Saiz, 2022). Examples of these processes for other groups of planktonic organisms are available. For instance, Padfield *et al.* (2016) and Barton *et al.* (2020) demonstrated that some species of planktonic microalgae were able to withstand the stress caused by warming after multiple generations of exposure and recovered previous carbon fixation rate levels. De Juan *et al.* (2023b), in a multigenerational study with the copepod *Paracartia grani* exposed to a temperature increase of 3 and 6°C, found a dampening of the warming effect on ingestion and egg production rates. Moreover, when reared for >18 generations, the warm-reared copepods had more elevated lethal temperatures and tolerance to heat stress pressure (de Juan *et al.*, 2023a, 2025). Thus, a deeper comprehension of the interaction between temperature and the organism’s behavior and physiological rates, whether in the short- or long-term, is necessary for a better understanding of their dynamics under a warming environment (Riemer *et al.*, 2018; Baltar *et al.*, 2019; Franzè and Menden-Deuer, 2020; van Moorsel *et al.*, 2023).

Apart from influencing at the individual level, temperature also drives interactions between species, causing an ecological impact: redistribution of species geographically and shifts in the dynamics of grazers and prey (Daufresne *et al.*, 2009; Caron and Hutchins, 2013; Gutow *et al.*, 2016). Warming is expected to affect the stability of the mixed layer, resulting in future scenarios of more severe and longer nutrient limitation for phytoplankton (Caron and Hutchins, 2013; Calbet *et al.*, 2015). This could have consequences for the microzooplankton, as they would have to face phytoplankton scarcity and even periods of starvation (Durant *et al.*, 2007; Caron and Hutchins, 2013; Anderson and Menden-Deuer, 2017). In this context, growing evidence suggests that changes in temperature and prey availability should be examined together, rather than independently, due to the potential for interactive effects and the varying strategies grazers employ to utilize food resources under different thermal conditions (Kimmance *et al.*, 2006; Huey and Kingsolver, 2019).

Predator–prey interactions are often assessed by examining functional and numerical responses to prey concentration, which describe, respectively, how many prey are consumed and how the grazer’s growth rate varies as a function of prey abundance. These ecophysiological responses become an interesting tool for predicting the repercussions of these interactions (Kiørboe *et al.*, 2018; Jeschke *et al.*, 2022). Consequently, understanding how temperature modulates both the ingestion and growth rates of the individuals depending on the available food could facilitate the comprehension of mismatches in predator–prey dynamics and changes in their relationship, possibly influencing predictions in food web models (Kimmance *et al.*, 2006; Durant *et al.*, 2007; Kiørboe *et al.*, 2018; Jeschke *et al.*, 2022).

In this study, we focused on the temperature response of the heterotrophic dinoflagellate *O. marina* feeding on the cryptophyte microalgae *R. salina* as prey. We determined the thermal performance curves of different lines of *O. marina* long-term reared at different thermal scenarios (16, 19 and 22°C), and then analyzed the changes in their feeding and growth rates using functional and numerical responses, respectively, and in their growth efficiency. Furthermore, given the current increase in frequency and magnitude of heat wave episodes (Garrabou *et al.*, 2022), we also studied the response of these long-term reared populations to acute stress (+3°C), assessing the effects on their thermal performance traits. To our knowledge, no study comparing the functional responses of long-term acclimated protozoa to several temperatures and those exposed to a sudden change in temperature (short-term acclimated) has been conducted to date. Therefore, we believe this comparison could be an interesting tool for understanding the response of marine protozoa to heat anomalies of different magnitudes. We hypothesize that exposure over a long period (years) to a temperature will result in a lessening of the thermal effects, whereas the simulated heat wave (acute stress) will result in a stronger response.

## METHODS

### Cultures

We used the heterotrophic dinoflagellate *O. marina*, a greatly employed species for laboratory investigations with protozoa (Kimmance *et al.*, 2006; Hantzsche and Boersma, 2010; Calbet *et al.*, 2013). The *O. marina* strain employed was ICM-ZOO-OM001 (size: 15–20 μm ESD), isolated by A. Calbet off NW Mediterranean coast in 1995. Since its isolation, this strain was kept in a temperature-controlled room (19°C), in 260 mL untreated tissue culture polyethylene flasks filled with autoclaved 0.1 μm filtered seawater, and fed *R. salina* (6–8 μm ESD), strain K-0294. The *R. salina* originated from the Scandinavian Culture Collection of Algae and Protozoa at the University of Copenhagen and kept growing in the laboratory for several years, growing exponentially in batch cultures in f/2 medium (Guillard, 1975). Both cultures were maintained at 19°C, with a salinity of 38, approximately 100 μE m^−2^ s^−1^ of white fluorescent lights, and a 10:14 hours light:dark cycle.

We created two new thermal lines of *O. marina* originating from the original 19°C culture. Thus, a batch of the *O. marina* culture was transferred to 16°C in June 2019 (53 months before the experiments were carried out) and another batch was transferred to 22°C in November 2020 (34 months before the experiments). These temperatures were selected to align with the range to thermal variation in the area of isolation of the grazer (Calbet *et al.*, 2001). These newly created lines of *O. marina* were reared under the same conditions described previously. We will henceforth refer to these lines as “long-term exposed” to their respective temperatures.

### Thermal performance curves

To evaluate the effects of temperature on the numerical response (growth rate) among the three long-term exposed *O. marina* lines, we carried out thermal performance curve assays. To do that, and for each line, we kept the grazers in 600 mL Pyrex bottles and fed them with *R. salina ad libitum* until reaching the grazer concentration necessary for preparing the assay suspensions (approximately 1000 cells mL^−1^). Afterward, we prepared a stock suspension for each long-term exposed *O. marina* line with a grazer:prey ratio of *ca*. 1:1000 starting with 800 000 prey mL^−1^ (*ca*. 28 800 μg C_prey_ L^−1^; Calbet and Saiz, 2022). The concentrations of grazer and prey were checked by means of a Beckman Coulter Multisizer IV particle counter (aperture tube 100 μm). The three suspensions were amended with 700 μL of f/2 working solution per liter (final concentration equivalent to f/10) to ensure algal growth. The range of temperatures to which we exposed the grazers was between 14 and 31°C in 3°C intervals except at the lower limit (14, 16, 19, 22, 25, 28 and 31°C). Therefore, once the suspensions were prepared, we filled seven 600 mL Pyrex bottles, taking care to fill them gradually in three to four steps, gently mixing the suspension between fillings and homogenously distributing it among bottles. The bottles were placed inside temperature-controlled baths, under diode lighting (25–30 μE m^−2^ s^−1^ measured in the water) with a 10:14 hours light:dark cycle. Light intensity and temperature were monitored using data loggers (HOBO, Onset) placed inside each bath. We allowed the grazers to acclimate to the experimental conditions for three days, providing sufficient time for physiological adjustment to the temperature (Calbet and Saiz, 2023). This setup also ensured that grazers remained in saturating food conditions during the acclimation period. Through that period, the bottles were gently turned upside down daily in order to prevent the settling of the prey. The prey was not preconditioned to the target temperature to avoid possible confounding factors, such as changes in size, nutritional quality, etc. (Hantzsche and Boersma, 2010; Calbet *et al.*, 2013), that could affect the functional responses.

After the three-day acclimation period to each temperature, we carried out the thermal performance curve assays under saturating food conditions. Most prey were consumed by the end of the conditioning period; consequently, the new experimental suspensions were composed predominantly of fresh *R. salina*. For this purpose, we readjusted the grazer and prey concentrations in the respective *O. marina* suspensions to a grazer:prey ratio of *ca*. 1:50, starting with 100 000 prey mL^−1^ (*ca*. 3600 μg C_prey_ L^−1^; Calbet and Saiz, 2022); according to previous data, these conditions would result, given the grazer abundance, in a reduction in prey concentration around 20–30% after the 24 hours of incubation (Calbet *et al.*, 2013, 2022; Calbet and Saiz, 2022, 2023). The new suspensions were amended with 700 μL of f/2 working solution per liter and then used to fill four 75 mL untreated culture polyethylene flasks for each experimental temperature. We allowed at least two hours for the stabilization of grazing rates to their food concentrations before measuring the cell concentration of the initial samples (Calbet *et al.*, 2013). One of the flasks was sacrificed to verify the initial concentrations of *O. marina* and *R. salina* with the Coulter particle counter. The remaining flasks were placed inside temperature-controlled baths at their corresponding incubation temperature, under diode lighting (25–30 μE m^−2^ s^−1^ measured in the water) with a 10:14 hours light:dark cycle monitored with data loggers (HOBO, Onset). During the experimentation time, prey was prevented from settling as previously explained. After *ca*. 24 hours, the experiment was terminated, and the concentration of flasks was measured as described previously.

### Functional responses and growth efficiency under long-term warming and acute heat stress

We compared the functional responses of the *O. marina* lines long-term exposed to their respective temperatures with those obtained after the organism experienced a 3°C heat stress, simulating a heat wave event (Calbet and Saiz, 2022). This thermal increase was within the tolerance range according to the evidence obtained previously from the thermal performance curves (see Results section). These treatments will be hereafter referred to as “HS+3°C.”

To conduct the functional response experiments, we kept the grazers in 600 mL Pyrex bottles and fed them with *R. salina ad libitum* until reaching the grazer concentration necessary for the experiments (approximately 2000 *O. marina* mL^−1^). Subsequently, the culture was divided into halves: one was placed in a bath at its normal growth temperature (long-term exposed experiment), while the other was submerged in a bath subjected to a 3°C increase (HS+3°C experiment). During the acclimation period (three days), we readjusted the grazer and prey concentrations daily to a grazer:prey ratio of *ca*. 1:25, starting with 50 000 prey mL^−1^ (*ca*. 1800 μg C_prey_ L^−1^; Calbet and Saiz, 2022). According to previous data, this concentration fell on the mid-point in the *O. marina* functional response, between saturation and starvation. The acclimation period lasted three days (enough to allow for physiological acclimation to temperature; Calbet and Saiz, 2023), after which we conducted the functional response and growth assays.

For the experiments, for each *O. marina* line, we prepared experimental suspensions with a grazer:prey ratio of *ca*. 1:100 starting with a prey concentration of 200 000 prey mL^−1^ (*ca*. 7400 μg C_prey_ L^−1^; Calbet and Saiz, 2022). Concurrently, we prepared suspensions of only prey at the same concentration to serve as a control for algal growth. All these suspensions (experimental and control) were amended with 875 μL of f/2 working solution per liter (equivalent to an f/8) to ensure algal growth. Then, the suspensions were consecutively diluted by half with temperature-acclimated filtered seawater to obtain a total of seven serially halved concentrations.

Once the diluted series was prepared, we filled three experimental and three control 75 mL untreated culture polyethylene flasks as previously explained. We allowed at least one hour for the stabilization of grazing rates to their food concentrations before measuring the initial cell concentration (Calbet *et al.*, 2013). For each concentration, one flask of both experimental and control treatments was sacrificed to verify initial concentrations. Part of the flask was processed through the Coulter counter to measure the initial prey concentration; the remaining sample was preserved with 2% (v/v) acid Lugol’s suspension for determining the number of grazers using an inverted microscope. The remaining flasks were placed inside a temperature-controlled bath at their corresponding incubation temperature, under diode lighting (60–70 μE m^−2^ s^−1^ measured in the water) with a 10:14 hours light:dark cycle monitored using data loggers (HOBO, Onset), and not allowing prey to settle as previously explained. After *ca*. 24 hours the experiment was terminated, and the concentration of experimental and control flasks preserved as described previously. Lugol samples were processed using either Sedgewick-Rafter slides when grazer concentrations exceeded 300 cells mL^−1^, or Utermöhl chambers (Utermöhl, 1958) when the concentration was below 300 cells mL^−1^.

### Thermal performance curve calculations

The growth rate of *O. marina* in the thermal performance incubations was computed as:


(1) \begin{equation*} \mu =\frac{\ln \left({C}_f/{C}_i\right)}{t} \end{equation*}

where *μ* is the instantaneous growth rate (d^−1^), *C_i_* and *C_f_* correspond to the *O. marina* concentrations (cells mL^−1^) at the beginning and end of the incubation, respectively, and *t* is its duration (d).

The curve fitting for each data set (i.e. growth rates for each of the three thermal lines) was performed using the “rTPC” package (Padfield *et al.*, 2021) in the statistical program R v. 4.4.1. (R Core Team, 2024). The best model was selected by first excluding models that did not fit the experimentally obtained data (in our case, those that did not allow negative rates above the optimal performance). Then, based on the global score of the Akaike Information Criterion (AIC), from the nine selected models, the one with the lowest AIC, which was suitable for all three curves and had consistency by visual inspection (i.e. no unnecessary curvilinearity or overfitting throughout the central part of the curve) was chosen. Finally, the thermal model by Lactin *et al.* (1995) appeared to fit our growth rate data better:


(2) \begin{equation*} \mu =\exp \left(a\times T\right)-\exp \left(a\times{T}_{\mathrm{max}}-\left(\frac{T_{\mathrm{max}}-T}{\Delta T}\right)\right)+b \end{equation*}

where *a* is a constant that determines the steepness of the rising portion of the curve, *T* is the test temperature (°C), *T*_max_ is the temperature at which the curve begins to decelerate beyond the optimum (°C), *ΔT* is the thermal safety margin (TSM) (°C) and *b* is a constant that determines the height of the overall curve. For each curve, we estimated the following key thermal performance traits: *μ*_max_ represents the estimated maximum rate; *T*_opt_ represents the temperature at which growth rate reaches its maximum value; *T*_breadth_ accounts for the range of temperatures where growth rate values varied between 80% below and above the value at *T*_opt_; *CT*_max_ corresponds to the temperature from the upper part of the curve at which growth rate was zero; and finally the TSM was determined as the difference between *CT*_max_ and *T*_opt_. The 95% confidence intervals for the above-mentioned parameters were obtained by means of the R package “car”, through residual resampling bootstrapping (Fox and Weisberg, 2019).

For determining which thermal adaptation model followed *O. marina* (Fig. 1), we first calculated the *E*_S_ (eV) for each curve in the rising side of the curve from the slope of the Arrhenius plots (ln rate vs 1/*T*, where *T* is the temperature in Kelvin degrees), thus *E*_S_ = −slope × *k*_B_, where *k*_B_ is the Boltzmann constant (8.6173 × 10^−5^ eV K^−1^). *Q*_10_ coefficients were computed as:


(3) \begin{equation*} {Q}_{10}=\exp \left(\frac{E_{\mathrm{S}}}{k_{\mathrm{B}}}\times \frac{10}{T_m^2}\right) \end{equation*}

where *T_m_* is the mean for the range of temperatures over which the experiments were carried out (Raven and Geider, 1988).

Then, we calculated the mean of the activation energies of the three thermal lines (*Ē*_S_) in the ascending phase and compared it to the global activation energy (*E*_L_) estimated from the Arrhenius plot of the optimal temperatures and maximum physiological rates of the individual curves (see Fig. 1).

### Heat stress experiment calculations

Clearance and ingestion rates in the incubations were calculated using Frost (1972) equations; per capita rates were computed using the average concentration of grazers in the incubation, respectively for each replicate, according to Heinbokel (1978). The growth rate of *O. marina* in the bottles was computed as described in equation (1).

The ingestion data of the functional responses were fitted to a Holling type II equation in order to calculate the maximum intake (*I*_max_) and half-saturation constant (*K*_m_):


(4) \begin{equation*} I=\frac{I_{\mathrm{m}\mathrm{ax}}\times C}{K_{\mathrm{m}}+C} \end{equation*}

where *I* is the ingestion rate (prey ind^−1^ d^−1^) and *C* is the average prey concentration. Ingestion rates were also converted to prey carbon units according to Calbet and Saiz (2022). The clearance rate data were fitted to the function:


(5) \begin{equation*} F=\frac{I_{\mathrm{m}\mathrm{ax}}}{K_{\mathrm{m}}+C} \end{equation*}

where *F* is the clearance rate (mL ind^−1^ d^−1^). The maximum clearance rate (*F*_max_) was calculated from ingestion data by means of the modification in equation (4) established by Helenius and Saiz (2017). When the extrapolation of the curve to obtain the maximum clearance rates resulted in unrealistically high values, we used instead the mean of the experimentally obtained maximum clearance values.

The comparison of *O. marina* growth rates (*μ*; d^−1^) among treatments was carried out only at the 50 000 prey mL^−1^ concentration, which was the concentration at which the cultures were acclimated during the three days prior to the experiment, as numerical responses for the rest of concentrations had not been allowed to condition to prey availability. The growth rates of *O. marina* cannot be easily estimated as increase in cell carbon because this grazer is morphologically extremely plastic and its cell volume is in large part the result of the cell vacuole content, not an actual increase in the grazer’s protoplasm (Calbet *et al.*, 2013). Therefore, we used as a proxy for the gross-growth efficiency for *O. marina* the quotient between the grazer doublings rate at the intermediate prey concentration (50 000 prey mL^−1^) and its respective feeding rate (expressed as micrograms of prey carbon ingested per grazer and day).

### Statistical analyses

The differences between the estimated thermal performance curve parameters were assessed by the lack of overlapping of the confidence intervals (Smith *et al.*, 2019). To evaluate the model of long-term acclimation followed by the species, the statistical difference between *Ē*_S_ and *E*_L_ was assessed by a t-test. If the species followed a “Hotter is better” model (Fig. 1A), the comparison between *Ē*_S_ and *E*_L_ would not be significant. Otherwise, if it followed a “Partial compensation” or “Hotter is partially better” model, *Ē*_S_ would be significantly greater than *E*_L_ (Fig. 1B). Finally, if *μ*_max_ did not vary with *T*_opt_ among the lines, then *E*_L_ would not be statistically different from zero and *O. marina* would be following a “Perfect or Complete compensation” model (Fig. 1C).

Data from the functional response and growth efficiency experiments with the long-term exposed lines were analyzed by two-way analysis of variance tests and post hoc Tukey tests when necessary. The normality of data was checked with Shapiro–Wilk test. A significance level of 0.05 was assumed to detect significant differences.

## RESULTS

### Thermal performance curves

**Fig. 2:**
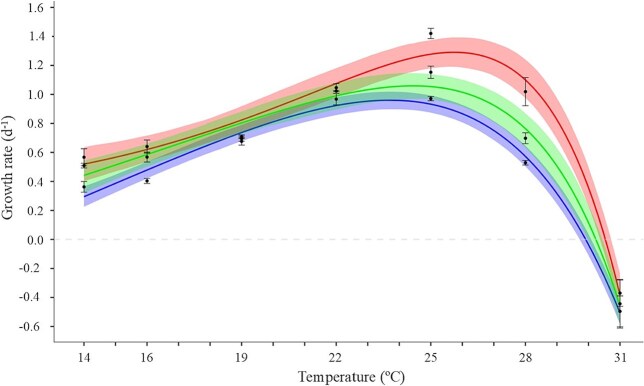
Thermal performance curves of the growth rate of *O. marina* long-term exposed at 16°C (blue), 19°C (green) and 22°C (red). Each point is the average for measurements of growth rate at the respective temperature (*n* = 3) and error bars are the standard error. The lines correspond to the best fitted model and the shaded regions represent the 95% confidence intervals estimated by residual resampling. The gray dashed line indicates zero growth.

The shape of the thermal performance curves of growth rate for the three long-term thermal lines of *O. marina* followed a typical unimodal, left-skewed shape (Fig. 2). There was a clear tendency for *μ*_max_ and *T*_opt_ to increase at higher long-term exposure temperatures, with significant differences between the 16 and the 22°C lines, and with the 19°C line showing intermediate values (Table I). The range of temperatures with high performances (*T*_breadth_) varied between 6.83 and 7.96°C of amplitude; it was lowest for the 22°C line, but did not differ from the other lines (Table I). Similarly to *μ*_max_ and *T*_opt_, the critical maximum temperatures (*CT*_max_) showed a positive relationship with temperature, increasing *ca*. 0.6°C between the 16 and the 22°C lines (Table I). Given the patterns in *CT*_max_ and *T*_opt_, the TSM decreased with temperature, with a statistically significant difference of 1.41°C between the two extreme thermal lines.

**Table I:** *Thermal performance traits of* O. marina *long-term thermal lines (16, 19 and 22°C)*.

Parameter	16°C Estimate (95% CI)	19°C Estimate (95% CI)	22°C Estimate (95% CI)
*μ*_max_ (d^−1^)	**0.96** (0.91–1.03)	**1.06** (0.97–1.13)	**1.29** (1.18–1.41)
*T*_opt_ (°C)	**23.70** (23.12–24.25)	**24.44** (23.42–25.28)	**25.74** (24.88–26.45)
*T*_breadth_ (°C)	**7.50** (7.17–7.84)	**7.96** (7.35–8.69)	**6.83** (6.14–7.55)
*CT*_max_ (°C)	**29.91** (29.72–30.09)	**30.24** (29.96–30.42)	**30.54** (30.37–30.73)
TSM (°C)	**6.21** (5.80–6.72)	**5.80** (5.04–6.67)	**4.80** (4.19–5.53)

*μ*_max_, maximum growth rate; *T*_opt_, optimal growth temperature; *T*_breadth_, range in which 80% of *μ*_max_ is reached; *CT*_max_, maximum critical temperature; TSM, thermal safety margin.

The activation energies for the 16, 19 and 22°C lines were respectively 1.07 ± 0.082SE, 0.67 ± 0.095SE and 0.77 ± 0.091SE eV, and showed statistically significant differences among them (*P* = 0.0079, covariance analysis). The corresponding *Q*_10_ coefficients were 4.30, 2.44 and 2.77, respectively.

The overall activation energy of the *O. marina* lines, *E*_L_, calculated from the *μ*_max_ and *T*_opt_ obtained for each thermal performance curve was 1.11 ± 0.036SE eV. This value proved not statistically different (t-test, *P* > 0.09) from the mean of the activation energies of each individual *O. marina* lines, *Ē*_S_, which was 0.84 ± 0.121SE eV.

### Functional responses

**Fig. 3:**
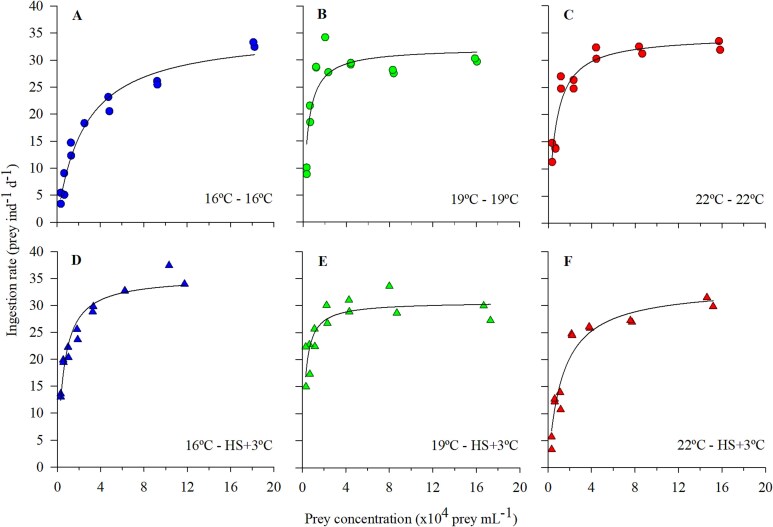
Cell-based ingestion rates as a function of prey concentration by (**A**, **D**) *O. marina* long-term reared at 16°C, (**B**, **E**) *O. marina* long-term reared at 19°C and (**C**, **F**) *O. marina* long-term reared at 22°C. Figures with circles correspond to long-term exposed individuals (**A**, **B**, **C**) and charts with triangles correspond to acute heat stress (HS+3°C) experiments (**D**, **E**, **F**). For each food concentration tested, replicate values are shown.

Long-term exposed individuals did not experience significant changes in their maximum cell intake (Fig. 3; Table II). All *O. marina* lines daily ingested between 32 and 35 cells per individual. When exposed to heat stress (HS+3°C), the ingestion rates of the three protozoan lines were not significantly affected. The same dynamics among long-term exposed populations and between long-term and their heat-stressed counterparts were observed when the maximum intakes were expressed in terms of carbon ingested (Supplementary Fig. S1; Table II).

**Table II:** *Functional response parameters (±SE) for the long-term exposed* O. marina *lines and for the acute heat stress (HS + 3°C) experiments*.

	16°C line		19°C line		22°C line	
Parameter	16°C	HS+3°C	19°C	HS+3°C	22°C	HS+3°C
*I*_max_ (prey ind^−1^ d^−1^)	**35.30** ^a^ ± 1.65	**35.33** ± 1.15	**32.22** ^a^ ± 1.79	**30.66** ± 1.30	**34.54** ^a^ ± 1.35	**33.58** ± 1.85
*I*_max_ (pg C_prey_ ind^−1^ d^−1^)	**1216** ^a^ ± 81.39	**1206** ± 49.98	**1100** ^a^ ± 56.40	**1190** ± 41.57	**1199** ^a^ ± 47.15	**1130** ± 74.11
*K*_m_ (prey mL^−1^)	**25 217** ^a^ ± 3588	**5554** ^*^ ± 750	**4003** ^b^ ± 1125	**2587** ± 672	**6426** ^b^ ± 1079	**13 173** ^*^ ± 2466
*K*_m_ (×10^5^ pg C_prey_ mL^−1^)	**5.33** ^a^ ± 1.24	**1.48** ^*^ ± 0.29	**1.04** ^b^ ± 0.31	**0.55** ± 0.17	**1.99** ^b^ ± 0.35	**2.74** ± 0.71
*F*_max_ (μL ind^−1^ d^−1^)	**1.44** ^a^ ± 0.08	**4.41** ^*^ ± 0.13	**3.28** ^b^ ± 0.18	**5.80** ^*^ ± 1.33	**3.79** ^b^ ± 0.56	**2.00** ± 0.05

*I*_max_, maximum intake; *K*_m_, saturation constant; *F*_max_, maximum clearance rate. Superscripts with letters and asterisks indicate the statistical significance of the comparison among the long-term exposed lines and between the long-term exposed and HS+3°C experiments, respectively. For all cases, significance was established when *P* < 0.05.

Although the maximum intake did not vary substantially with respect to temperature, *K*_m_ values, both expressed in prey number and carbon, underwent significant changes. The *K*_m_ of the 16°C line, expressed in cells, was 4–6 times higher than that of the other two long-term lines (Fig. 3; Table II); in terms of carbon the pattern was similar (Supplementary Fig. S1; Table II). When exposed to heat stress (+3°C), both the *K*_m_ in cell and carbon decreased in the 16 and 19°C lines, although differences were only significant for the 16°C line (Fig. 3 and Supplementary Fig. S1; Table II). The 22°C line showed an opposite pattern, with an increase in the *K*_m_ values (Fig. 3 and Supplementary Fig. S1; Table II).

**Fig. 4:**
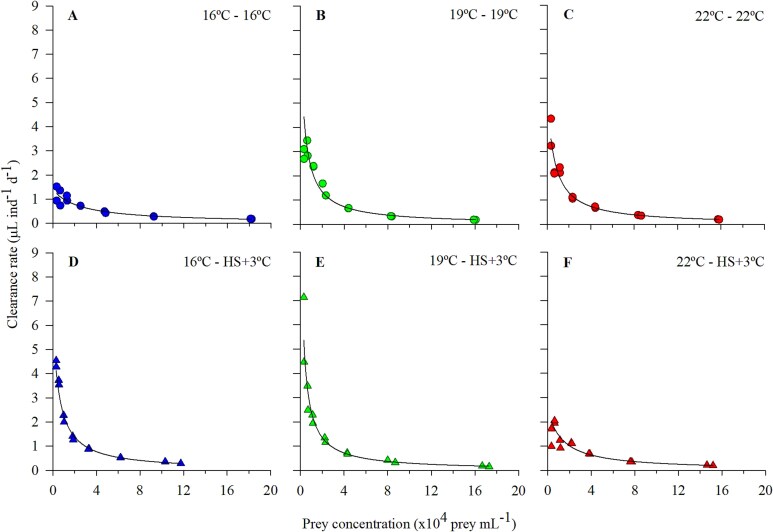
Clearance rates as a function of prey concentration by (**A**, **D**) *O. marina* long-term reared at 16°C, (**B**, **E**) *O. marina* long-term reared at 19°C and (**C**, **F**) *O. marina* long-term reared at 22°C. Figures with circles correspond to long-term exposed individuals (**A**, **B**, **C**) and charts with triangles correspond to acute heat stress (HS+3°C) experiments (**D**, **E**, **F**). For each food concentration tested, replicate values are shown.

Finally, regarding the clearance rate (Fig. 4), there was an overall positive relationship between temperature and the maximum clearance rate among the three thermal lines, with statistical differences among the 16 and 22°C lines (Table II). In the HS+3°C experiments, on the other hand, the 16 and 19°C lines exhibited a notable, statistically significant, increase in clearance rate, while the 22°C +3°C line showed an opposite pattern, almost halving the value.

### Effects of temperature on growth rate and growth efficiency

**Fig. 5:**
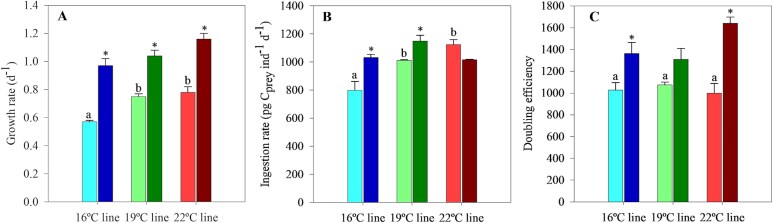
Growth rate (**A**), ingestion rate (**B**) and doubling efficiency (**C**) for the three *O. marina* thermal lines. Light bars correspond to long-term exposed individuals and dark bars correspond to acute heat stress (HS+3°C) experiments. Doubling efficiency was calculated as the quotient between the doubling rate (calculated from growth rate) and the ingestion rate. Note that all the rates were determined at a concentration of 50 000 prey mL^−1^, which was the acclimation prey concentration for all treatments. Superscripts with letters and asterisks indicate the statistical significance of the comparison among the long-term exposed lines and between the long-term exposed and HS+3°C experiments, respectively. For all cases, significance was established when *P* < 0.05. The error bars are the standard error.

There were significant differences in the growth rate among the long-term exposed lines, with the growth rate of long-term exposed *O. marina* at 16°C being 25% lower than those at 19 and 22°C (Fig. 5A). The +3°C heat stress resulted in a significant increase in growth rates for all lines, with increments ranging between 39 and 70% (Fig. 5A). The ingestion rates of *O. marina* at 50 000 prey mL^−1^ (Fig. 5B) showed a tendency to increase among long-term exposed lines; heat stress also had a positive stress on feeding rates, except for the 22°C line (Fig. 5B). Regarding the doubling efficiency, we found no significant differences among the three long-term exposed thermal lines (Fig. 5C). In the HS+3°C experiments, however, warming caused an increase in the efficiency of the 16°C and particularly the 22°C line; in the 19°C line, on the other hand, the increase was not statistically significant (Fig. 5C).

## DISCUSSION

### *O. marina*’s adaptation model

This study provided insights into the thermal tolerance of the heterotrophic dinoflagellate *O. marina*, focusing on its performance under varying temperature regimes, including long-term acclimation and short-term exposure. According to the thermal performance curves only, our study could suggest that this protozoan would follow a “Hotter is better” model of adaptation, as described in previous studies (Frazier *et al.*, 2006; Barton and Yvon-Durocher, 2019; Smith *et al.*, 2019; Chen *et al.*, 2023). The overall activation energy calculated from the *μ*_max_ and *T*_opt_ of the three thermal lines was not different from the mean of the activation energies of the three of them. Accordingly, maximum growth (*μ*_max_) and optimal temperature (*T*_opt_) tended to increase with rising acclimation temperature. Moreover, the asymmetry of the curves (left-skewed) and the better performance at higher temperatures align well with the findings of Martin and Huey (2008), who stated that maximum yield in thermal performance curves should be above the rearing temperature because of Jensen’s inequality (i.e. the average of the performances of the individual at varying temperatures is not equal to the performance at the average of those temperatures; Olivares *et al.*, 2022) and the asymmetry of the curve, maximizing fitness at warmer temperatures. This would imply that strains reared at high temperatures may perform more optimally and be advantageous in warming scenarios after thermal selection (Malusare *et al.*, 2023). Apart from this, the narrowing of thermal amplitude (*T*_breadth_) with increasing long-term acclimation temperature may reflect specialization to more constrained thermal conditions (Knies *et al.*, 2009). However, while *O. marina* long-term exposed at 16°C showed a null growth rate at 29.9°C (Table I), the 19 and 22°C lines continued experiencing positive growth rates at that temperature (Fig. 2). This would confer an evolutive fitness advantage to the protozoan toward climate warming: populations with higher *CT*_max_ acquired more heat tolerance and would be able to cope with severe acute warming, within their thermal limits (at least for the time period tested in our study; de Juan *et al.*, 2023a).

Functional responses corroborated the influence of temperature on *O. marina*’s physiological traits. Within tolerance limits, growth is generally accelerated with warming, consistent with the general relationship between metabolism and temperature. In the case of heterotrophs, respiration is the metabolic trait most likely to be impacted (López-Urrutia *et al.*, 2006). Consequently, due to the pressure exerted by temperature, the organism should balance its metabolic needs and food ingestion for ensuring the population’s survival under different thermal scenarios (Huey and Kingsolver, 2019; de Juan *et al.*, 2025), particularly at lower prey concentrations (Okuyama, 2010; Daugaard *et al.*, 2019). In the case of *O. marina*, the dinoflagellate increased its clearance rate considerably when exposed to higher temperatures, likely due to an increase in swimming activity, which would positively affect the encounter rates with prey (Kiørboe and Saiz, 1995). The increase in clearance rate also resulted in a diminution in the half-saturation constant, reaching satiation at significantly lower prey abundances.

Interestingly, between the 19 and 22°C lines, growth and feeding responses showed no significant differences, and doubling efficiencies remained stable across thermal lines (Fig. 5). This stability suggests adaptive adjustments and partial thermal compensation for energetic demands after multigenerational exposure (Seebacher *et al.*, 2015; Havird *et al.*, 2020). Similarly, the maximum prey intake rates were maintained across all thermal lines, which also indicates the existence of compensatory mechanisms in *O. marina*. Altogether, these observations seem to indicate that instead of a pure “Hotter is better” framework, as suggested by the analysis of only the thermal performance assays, *O. marina* would align more closely with a “Hotter is partially better” adaptation model.

The “Hotter is partially better” hypothesis postulates an improvement of physiological traits with temperature, similarly to the “Hotter is better” model. However, while colder temperatures constrain metabolic reactions (leading to reduced growth and clearance rates and a higher half-saturation constant), warmer temperatures may not always enhance physiological rates indefinitely. This is because, beyond a certain physiological threshold, the species may struggle to cope with metabolic demands, resulting in a decline in performance (Luhring and DeLong, 2017; Kontopoulos *et al.*, 2020). This could be the case for our 19 and 22°C populations, which exhibited similar activation energies and *Q*_10_ coefficients, indicating they were closer to their optimal temperatures and thus more constrained by further warming. The “Hotter is partially better” hypothesis acknowledges that adaptation and multigenerational exposure to thermal stress allow the protozoans to adjust their metabolism and partially overcome thermodynamic constraints, thereby enabling them to inhabit a particular thermal niche over an extended period (Barton and Yvon-Durocher, 2019; Havird *et al.*, 2020; Liu *et al.*, 2022). The partial compensatory behavior shown by *O. marina* may reveal the high active plasticity of this dinoflagellate (Schulte *et al.*, 2011; Havird *et al.*, 2020), possibly due to its natural habitat in coastal areas, particularly in intertidal pools, estuaries and marshes (Watts *et al.*, 2011). These environments can experience great temperature fluctuations throughout the day, more than a 10°C variation (Smit and Glassom, 2017). That behavior would confer *O. marina* an ecological advantage over other species that do not have this compensatory capacity, and its population would persist in the face of this type of environmental variation.

### Resilience of *O. marina* to acute heat stress and sublethal temperatures

Acute heat stress experiments highlighted *O. marina*'s capacity to withstand sudden warming. Higher doubling efficiencies under heat stress compared to long-term exposure could be a result of increases in both ingestion and, particularly, growth rates (Fig. 5). The 22°C line, being closer to its optimal growth temperature, displayed particularly high doubling efficiency. Indeed, *O. marina* may maximize reproduction despite the potentially higher metabolic costs associated with heat stress. Similar strategies have been observed for other zooplankters under heat stress scenarios (de Juan *et al.*, 2023b, 2025). Maximizing reproduction (i.e. growth rate) involved a reduction in *O. marina* cell size under long-term warming and when exposed to acute heat stress (Supplementary Fig. S2), possibly attributed to several factors encompassed by the Temperature-Size Rule (TSR; Atkinson, 1994; Atkinson *et al.*, 2003). According to the TSR, increased temperatures accelerate metabolic and doubling rates, causing protozoan organisms to complete their cell cycle more quickly, resulting in smaller final cell sizes. In our case, the magnitude of the decline in cell volume was approximately 600 μm^3^ cell^−1^ between exposure temperatures, i.e. 2.5% °C^−1^, as previously reported for protists (Atkinson *et al.*, 2003). Under heat stress, the demands of population growth become more sensitive to temperature than to prey ingestion (Tabi *et al.*, 2020), leading to smaller cells as a plastic (non-adaptive) response.

The increase in doubling efficiency under heat stress implies the improvement of cellular metabolism and physiological activity without severe damage, as the result of the development of an acclimation response within thermal limits (Marchand *et al.*, 2005; Martin and Huey, 2008; Huey and Kingsolver, 2019). However, the decrease of clearance rate in the 22°C line under acute heat stress (+3°C) might imply that the species was starting to experience incipient detrimental effects in its physiology (“metabolic meltdown”; Huey and Kingsolver, 2019), as similarly observed in previous studies (Kimmance *et al.*, 2006). Under severe stress conditions, organisms may carry out behavioral or physiological adjustments to reduce energy demands and maintain homeostasis (Angilletta *et al.*, 2003; Enzor *et al.*, 2017; Zhao *et al.*, 2017). Thus, one of the first adjustments planktonic organisms may develop to avoid metabolic meltdown would involve a reduction in motility (swimming activity), which implies lower clearance rates (Huey and Kingsolver, 2019). Thus, behavioral and physiological mechanisms involved in coping with the thermal stress response may benefit some traits, such as growth rate, while imposing costs on others, such as clearance rate (Angilletta *et al.*, 2003). Previous thermal history and time exposure to the tested temperature are therefore crucial for evaluating the response of individuals to environmental change (Havird *et al.*, 2020).

Thermal performance curves revealed that once the *T*_opt_ was surpassed, the growth rate quickly declined across all thermal lines. Furthermore, the TSM of the species experienced a decrease with higher long-term exposure temperature, indicating a narrower buffer capacity against thermal extremes (Gunderson and Stillman, 2015; Seebacher *et al.*, 2015). Lines reared at higher temperatures would be more affected by small changes in temperatures above *T*_opt_, reflecting an adaptive trade-off (Montagnes *et al.*, 2022), as perceived in the 22°C line for its clearance rate. Contrary to Logan and Cox (2020) assertion that upper thermal limits are more readily subject to evolutionary change, Montagnes *et al.* (2022) proposed a different sequence of adaptation. They suggested that, under selective pressure, an organism's optimal temperature (*T*_opt_) evolves first to align with the local average temperature, thereby enabling the organism to adapt effectively to its specific environmental niche. Afterwards, *CT*_max_ might evolve to higher temperatures to extend the thermal tolerance of the organism. These parameters are highly dependent on the environment, even changing among strains of the same species: e.g. the *O. marina* strains from the studies of Franzé and Menden-Deuer (2020) (isolated from Salish Sea coast) and ours (isolated from NW Mediterranean coast) share a similar value for *CT*_max_, whereas Jung *et al.* (2021) observed positive growth rates for its *O. marina* strain (isolated from Korean coast) even at 32°C. This trait may provide them with local adaptive qualities, broadening their thermal limits to cope with thermal stresses in their niche.

In this regard, it is important to notice that our *O. marina* strain was isolated and maintained in the laboratory at 18–19°C since 1995, then transferred to 16°C (more than four years before performing the experiments) and finally to 22°C (almost three years before the analyses). The time required for protozoa to adapt to a new temperature condition is not well established (Franzé and Menden-Deuer, 2020; Calbet and Saiz, 2022). However, for phytoplankton species, about 100 generations are needed to observe compensatory temperature effects (Padfield *et al.*, 2016; Barton *et al.*, 2020, 2023), or even less (Brand *et al.*, 1981). Assuming that *O. marina* adapts at a rate similar to phytoplankton, and given the time spent at each temperature, the number of generations in our cultures appeared sufficient to ensure proper thermal adaptation in the long-term exposures.

## CONCLUSIONS

*O. marina* is a widely distributed dinoflagellate whose presence has been observed in numerous locations, particularly some with very high diel thermal ranges (e.g. tide pools; Hantzsche and Boersma, 2010). Therefore, it should be able to make physiological adjustments to withstand changes in environmental conditions, as demonstrated by the partial compensatory effects exhibited under long-term exposure and the wide *T*_breadth_. This response may be more limited in other, more oceanic, protozoan species. However, acute thermal stress imposed physiological costs, such as reduced clearance rates, as observed in the 22°C line when exposed to 25°C. These limitations could challenge *O. marina*’s ability to thrive under sustained warming and episodic heat waves, which are becoming more frequent due to climate change (Hays *et al.*, 2005; Garrabou *et al.*, 2022).

As anticipated, long-term exposure (years) to temperature allowed for thermal compensation of metabolism, whereas acute heat stress triggered more significant responses in certain physiological variables. We acknowledge that our studies are conducted with long-term constant temperatures and controlled conditions, which could not accurately predict the behavior of the protozoa in natural environments (Schulte *et al.*, 2011). In their natural habitats, these organisms experience seasonal fluctuations in water temperature and increasingly frequent and intense heatwaves, which could pose greater risks to their physiology and intensify their vulnerability.

Additionally, global change factors rarely occur in isolation; organisms will also face other environmental stressors, such as nutrient limitation or prey changes in size and abundance, which could affect their performance and ecological functions. Despite the importance of understanding how heat events interact with variables like food availability and quality, such combined effects remain poorly studied. Therefore, studies simulating increasing chronic warming along with sudden heat waves and in combination with other biotic or abiotic factors can help to better understand the vulnerability threshold and resilience of protozoa, as well as the adaptive processes they may develop under those conditions.

This research is critical for improving predictions related to trophic interactions and biogeochemical processes in the context of global climate change. Given the projected rise in global ocean temperatures and the increased frequency of extreme events, understanding the adaptive strategies of *O. marina* extends beyond academic curiosity—it has direct implications for marine ecosystem resilience and biogeochemical cycles. The ability of this species, and others, in the microbial food web to adapt and thrive under changing conditions will be vital for maintaining energy transfer and nutrient cycling within marine food webs. Consequently, addressing these knowledge gaps is essential for accurately predicting and managing the impacts of climate change on ocean ecosystems.

## Supplementary Material

Supplementary_data_fbaf013

## Data Availability

The dataset will be available in the DIGITAL.CSIC repository upon publication of the manuscript.
