# Supplementary material for: Thermal performance of the heterotrophic dinoflagellate Oxyrrhis marina under long-term warming and acute heat stress
Source: J Plankton Res. 2025 Aug 12;47(5):fbaf013. doi: 10.1093/plankt/fbaf013 (PMC12343005; doi:10.1093/plankt/fbaf013)
Supplement: Supplementary_data_fbaf013 [file supplementary_data_fbaf013.docx]

**SUPPLEMENTARY DATA**


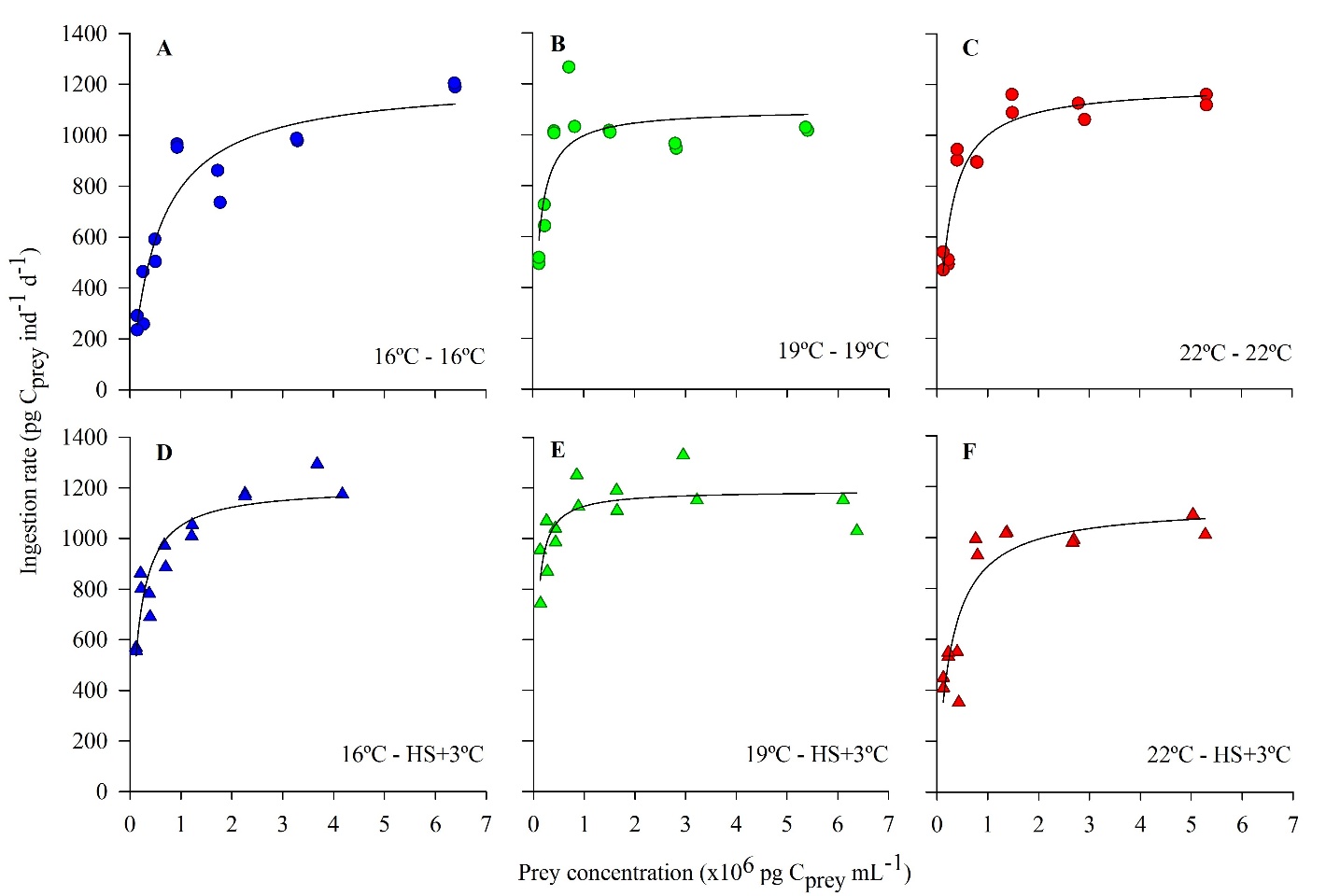


**Fig. S1.** Carbon-based ingestion rates as a function of prey concentration by (**A**, **D**) *O. marina* long-term reared at 16ºC, (**B**, **E**) *O. marina* long-term reared at 19ºC and (**C**, **F**) *O. marina* long-term reared at 22ºC. Figures with circles correspond to long-term exposed individuals (**A**, **B**, **C**) and charts with triangles correspond to acute heat stress (HS+3ºC) experiments (**D**, **E**, **F**). For each food concentration tested, replicate values are shown.


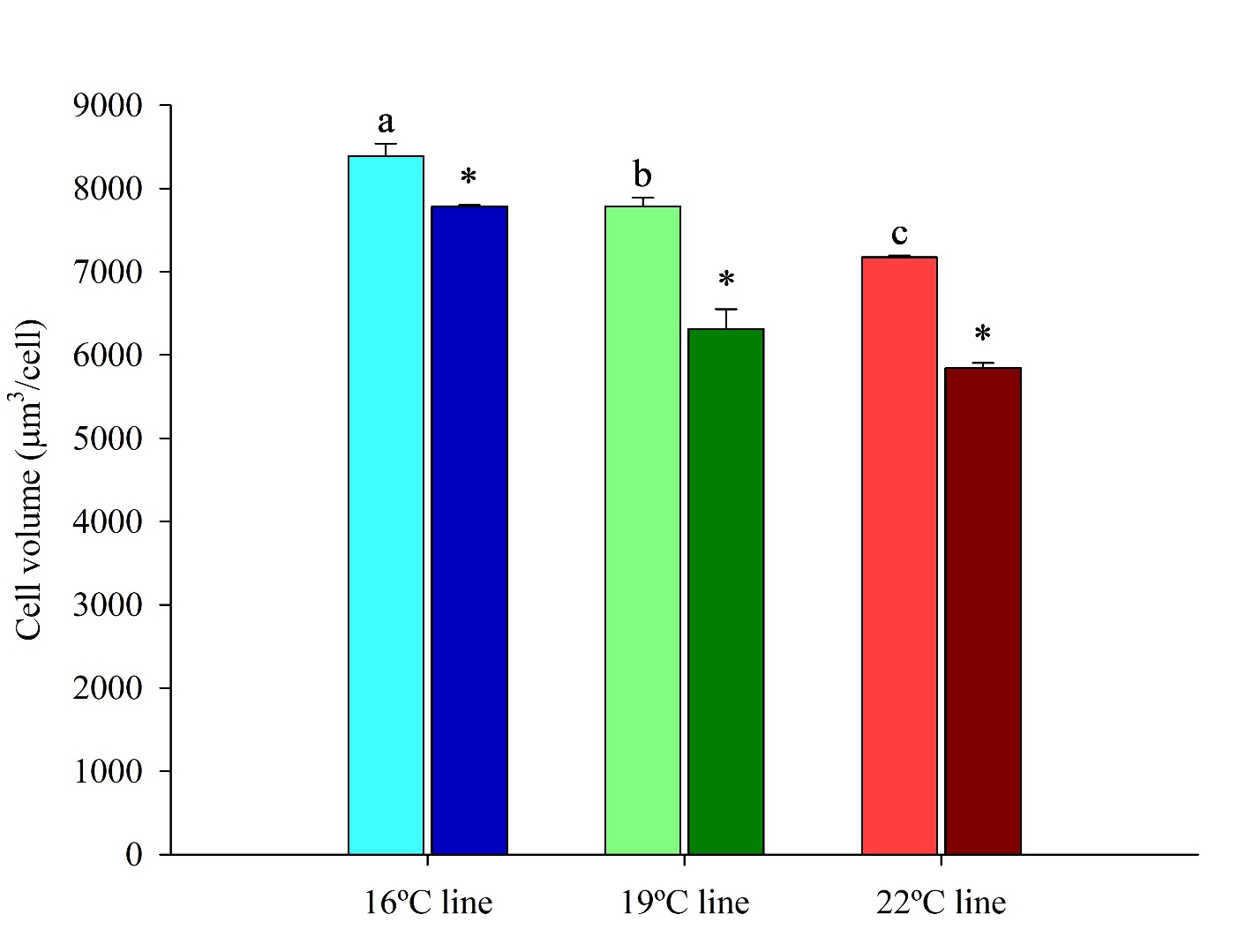


**Fig. S2.** Cell volume as proxy of cell size for the three *O. marina* thermal lines. Light bars correspond to long-term exposed individuals and dark bars correspond to acute heat stress (HS+3ºC) experiments. All the cell volumes were determined at the highest prey concentration (200,000 prey mL^-1^) after 24 hours of experimental incubation period, for ensuring complete saturation of the cells. Superscripts with letters and asterisks indicate the statistical significance of the comparison among the long-term exposed lines and between the long-term exposed and HS+3ºC experiments, respectively. For all cases, significance was established when *p*<0.05. The error bars are the standard error.
